# Evaluation of cognitive functions, emotional disturbances and acceptance of the disease in patients with cardiovascular disorders and type D personality

**DOI:** 10.1192/j.eurpsy.2023.857

**Published:** 2023-07-19

**Authors:** M. Piegza, L. Kunert, P. Dębski, K. Podkowska – Kurpas, A. Szczecina, A. Leksowska, J. Piegza, P. Gorczyca

**Affiliations:** 1 Department of Psychiatry, Faculty of Medical Sciences in Zabrze; 2 Department of Psychoprophylaxis, Faculty of Medical Sciences in Zabrze; 3 Students Scientific Association by the Department of Psychiatry, Faculty of Medical Sciences in Zabrze, Medical University of Silesia in Katowice, Tarnowskie Góry; 4 Third Department of Cardiology, Faculty of Medical Sciences in Zabrze, Medical University of Silesia in Katowice, Zabrze, Poland

## Abstract

**Introduction:**

The majority of people with cardiovascular disorder meets the criteria of type D personality. Its prevalence, however, favours experiencing negative emotions and avoiding social connections [Kupper et al. Int J Cardiol. 2013;166(2) 327-33]. Cardiovascular disorders’ steady morbidity growth entitles to search for the factors, which have an impact on functioning, acceptance of the disorder and obeying doctor’s orders among patients with this diagnosis [Leu et al. J Formos Med Assoc. 2019;118(3) 721-729]. One of the factors, which largely determines mental efficiency, except for anxiety and depression symptoms, is cognitive functioning [Burkauskas et al. Cogn Behav Neurol.2016;29(2)91-9, Schiffer et al. Eur J Heart Fail. 2008;10(8) 802-10].

**Objectives:**

Evaluation of cognitive functioning, acceptance of the disorder, intensifying of anxiety and depression symptoms among people who suffer from cardiovascular diseases with type D personality and seeking for relationships between those parameters.

**Methods:**

102 people took part in the study, including 63 men and 39 women, the average age amounting to 65,471 (SD±10,567). Patients were divided according to the presence of type D personality, gender and cardiological diagnoses. The DS-14 scale was used to assess the type D personality, the HADS scale to assess the symptoms of anxiety and depression, and also the AIS scale to assess the acceptance of the disease and MoCA 7.2 scale for cognitive functions. The original questionnaire was used to collect the necessary sociodemographic data, data on the type and course of the main disease, comorbidities and medications taken.

**Results:**

About 37% of respondents meet the criteria of type D personality. The AIS scores correlate negatively with age, disease duration, and with both components of the DS-14 scale (negative emotions-Ne and social inhibition-Hs). Both DS-14 subscales correlate positively with HADS-A and HADS-D, and the DS-14 (Ne) subscale is also positively associated with age. The results of the MoCA scale negatively correlate with age and duration of the disease. People without personality traits of type D have higher AIS scores, lower HADS-A (fig.1) and HADS-D scores (fig.2), and higher MoCA scores (fig.3) than those with type D personality. There were no differences between patients with ischemic heart disease and patients with ischemic heart disease and heart failure. In the subscale of social inhibition DS-14 (Hs), women obtained a higher result.

**Image:**

**Figure:**
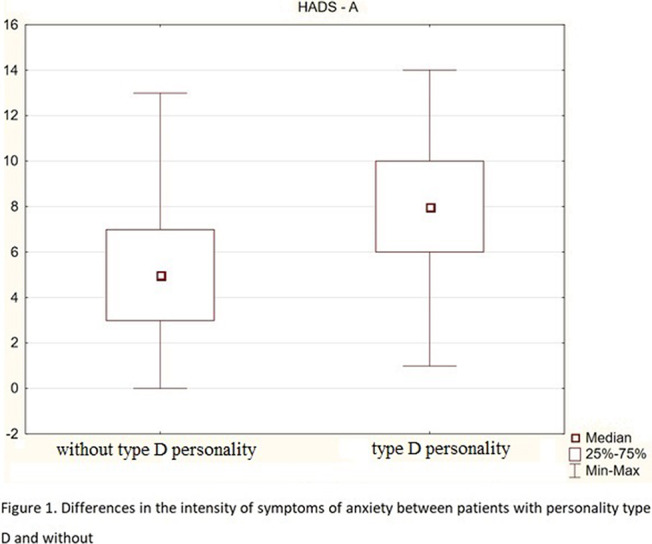

**Image 2:**

**Figure:**
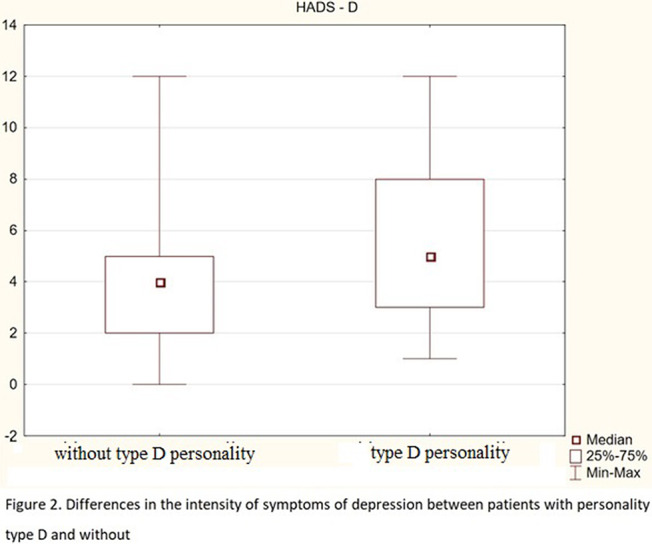

**Image 3:**

**Figure:**
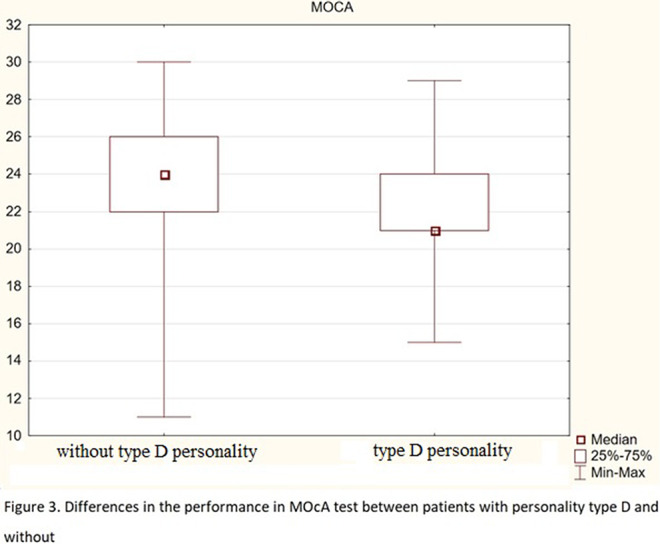

**Conclusions:**

1. People with D personality are more difficult to accept their illness, they are characterized by a higher level of depression and anxiety, and weaker cognitive functions.

2. Women are characterized by stronger social inhibition.

3. Younger people with a shorter medical history accept the disease more easily.

4. Heart failure is not a factor differentiating the studied group of patients.

**Disclosure of Interest:**

None Declared

